# Autoimmune versus Non-autoimmune Cutaneous Features in Monogenic Patients with Inborn Errors of Immunity

**DOI:** 10.3390/biology12050644

**Published:** 2023-04-24

**Authors:** Niusha Sharifinejad, Gholamreza Azizi, Seyed Erfan Rasouli, Zahra Chavoshzadeh, Seyed Alireza Mahdaviani, Marzieh Tavakol, Homa Sadri, Mohammad Nabavi, Sareh Sadat Ebrahimi, Afshin Shirkani, Ahmad Vosughi Motlagh, Tooba Momen, Samin Sharafian, Mehrnaz Mesdaghi, Narges Eslami, Samaneh Delavari, Sasan Bahrami, Reza Yazdani, Nima Rezaei, Hassan Abolhassani

**Affiliations:** 1 Non-Communicable Diseases Research Center, Alborz University of Medical Sciences, Karaj 3149969415, Iran; niushasharifinejad@gmail.com (N.S.);; 2 Research Center for Immunodeficiencies, Pediatrics Center of Excellence, Children’s Medical Center, Tehran University of Medical Sciences, Tehran 1419733141, Iran; 3 Pediatric Infections Research Center, Mofid Children’s Hospital, Shahid Beheshti University of Medical Sciences, Tehran 1985717443, Iran; 4 Pediatric Respiratory Diseases Research Center, National Research Institute of Tuberculosis and Lung Diseases, Shahid Beheshti University of Medical Sciences, Tehran 1985717443, Iran; 5 Department of Allergy and Clinical Immunology, Rasool e Akram Hospital, Iran University of Medical Sciences, Tehran 1449614535, Iran; 6 Department of Immunology and Allergy, Kerman University of Medical Sciences, Kerman 7619833477, Iran; 7 Allergy and Clinical Immunology Department, School of Medicine, Bushehr University of Medical Science, Moallem St., Bushehr 7514763448, Iran; 8 Department of Pediatrics, North Khorasan University of Medical Sciences, Bojnurd 7487794149, Iran; 9 Department of Asthma, Allergy and Clinical Immunology, Child Growth and Development Research Center, Research Institute of Primordial Prevention of Non-Communicable Disease, Isfahan University of Medical Sciences, Isfahan 8174673461, Iran; 10 Department of Digital Media, Westphal College of Media Arts and Design, Drexel University, Philadelphia, PA 19104, USA; 11 Division of Clinical Immunology, Department of Biosciences and Nutrition, Karolinska Institutet, Karolinska University Hospital, Huddinge, 141 86 Stockholm, Sweden

**Keywords:** inborn errors of immunity, primary immunodeficiency diseases, cutaneous manifestations, autoimmune cutaneous manifestations, skin infections

## Abstract

**Simple Summary:**

Cutaneous manifestations were observed in ~44% of Iranian patients with monogenic IEI. A considerable number of these patients developed cutaneous disorders as their first manifestation of the disease. Skin disorders in IEI patients might delay the immunodeficiency diagnosis but may improve their prognosis.

**Abstract:**

Cutaneous manifestations are one of the most common presentations among patients with inborn errors of immunity (IEI). These skin manifestations are often among the first presenting features in the majority of patients preceding the IEI diagnosis. We studied 521 available monogenic patients with IEI listed in the Iranian IEI registry up to November 2022. We extracted each patient’s demographic information, detailed clinical history of cutaneous manifestations, and immunologic evaluations. The patients were then categorized and compared based on their phenotypical classifications provided by the International Union of Immunological Societies. Most patients were categorized into syndromic combined immunodeficiency (25.1%), non-syndromic combined immunodeficiency (24.4%), predominantly antibody deficiency (20.7%), and diseases of immune dysregulation (20.5%). In total, 227 patients developed skin manifestations at a median (IQR) age of 2.0 (0.5–5.2) years; a total of 66 (40.7%) of these patients initially presented with these manifestations. Patients with cutaneous involvement were generally older at the time of diagnosis [5.0 (1.6–8.0) vs. 3.0 (1.0–7.0) years; *p* = 0.022]. Consanguinity was more common among patients who developed skin disorders (81.4% vs. 65.2%, *p* < 0.001). The overall skin infection rate and the type of dominant pathogens were significantly different among the IEI patients in different phenotypical classifications (*p* < 0.001). Atopic presentation, including urticaria, was highly prevalent among patients with congenital defects of phagocytes (*p* = 0.020). The frequency of eczema was also significantly higher among cases with both syndromic and non-syndromic combined immunodeficiency (*p* = 0.009). In contrast, autoimmune cutaneous manifestations, including alopecia and psoriasis, were most common in patients with immune dysregulation (*p* = 0.001) and defects in intrinsic or innate immunity (*p* = 0.031), respectively. The presence of autoimmune cutaneous complications significantly improved the survival rate of IEI patients (*p* = 0.21). In conclusion, cutaneous manifestations were observed in nearly 44% of Iranian patients with monogenic IEI. A considerable number of patients with cutaneous involvements developed these disorders as their first manifestation of the disease, which was particularly noticeable in patients with non-syndromic combined immunodeficiency and phagocytic defects. The neglected skin disorders in IEI patients might delay diagnosis, which is generally established within a 3-year interval from the development of skin-related problems. Cutaneous disorders, especially autoimmune features, might indicate a mild prognosis in IEI patients.

## 1. Introduction

Inborn errors of immunity (IEI), previously known as primary immunodeficiencies, are a group of heterogeneous rare diseases with a wide range of clinical presentations [1]. Cutaneous involvements are a common manifestation that have been reported in 30–70% of patients with IEI [2,3,4]. These manifestations are among the presenting features in the majority of patients preceding IEI diagnosis [5]. Although skin infections characterize many disorders in this category, some patients may also manifest non-infectious cutaneous signs, specific dermatologic complications, or even malignancy [1].

So far, few studies have investigated cutaneous manifestations in patients with IEI; only one of them originated from Iran and it involved a limited number of patients [2,3,4,6]. These manifestations mainly ranged from infections (especially bacterial) and eczematoid erythroderma to alopecia and petechia/purpura [3,4,6]. Therefore, comprehensive information regarding this aspect of IEI is quite scarce and the molecular defects underlying these manifestations are not well-reported. Further investigations could clarify the type of skin disorders in IEI and whether these manifestations could serve as a warning sign of an underlying immunodeficiency.

Thus, we conducted a comprehensive retrospective evaluation of infectious and non-infectious cutaneous involvements in a considerable number of Iranian patients with monogenic IEI. Furthermore, we investigated whether these skin manifestations were primary or secondary presentations with specific consideration of autoimmune manifestations. This study elaborated on the importance of cutaneous manifestations in IEIs and their diagnostic value for clinical immunologists.

## 2. Patients and Method

### 2.1. Patients

This retrospective study was conducted on a cohort of patients with IEI documented in the Iranian IEI registry [7,8] up to November 2022. The database is located in the Research Center for Immunodeficiencies, Children’s Medical Center, Tehran, Iran; this facility is a referral center for suspected or diagnosed IEI cases catering to patients from all over Iran. Before data collection, written informed consent was obtained from each patient and/or their legal guardians. This study was approved by the Ethics Committee of Tehran University of Medical Sciences (IR.ABZUMS.REC.1401.229).

### 2.2. Data Collection

The patients were primarily diagnosed and managed according to the criteria of the European Society for Immunodeficiencies (ESID) [9] and the Middle East and North Africa Diagnosis and Management Guidelines for IEI [10]. A proper questionnaire surveyed the patients’ demographic information, including sex, age of disease onset, age of diagnosis, age of developing cutaneous disorders, time of follow-up, life status, family history, and detailed clinical history of cutaneous manifestations. The evaluation for cutaneous involvements was reviewed for all patients by an immunologist and a dermatologist. The skin lesions were re-evaluated with the recently published atlas of IEI clinical manifestations [11]. Laboratory evaluations were collected, including complete blood and differential counts, serum immunoglobulin levels, and flow cytometric evaluation of lymphocyte subsets, as explained previously [8].

### 2.3. Genetic Analysis

Genomic DNA was extracted from the available patients’ whole blood samples. Depending on whether they had the classical clinical presentations suggestive of a specific IEI, targeted or whole-exome sequencing was performed on patients using a previously described pipeline [12,13,14,15]. Sequences were generated and compared to the human genome reference (UCSC hg 19 version; build 37.1) after raw image file processing using the BWA mem software (0.7.10-r789), while Picard MarkDuplicates (v1.117) discerned the duplicated reads. To minimize the number of mismatching bases, GATK RealignerTargetCreator and GATK IndelRealigner (v3.3-0) were used to control the resulted BAM file. The GATK HaplotypeCaller and GATK GenotypeGVCFs (v3.3-0) were subsequently used to call genotypes in target regions, with 30.0 chosen as the minimum Phred-scaled confidence threshold at which variants were called. For analysis of WES, we followed a previously described protocol for prioritizing candidate variants—homozygosity mapping— predicting their effect on protein, large deletion, and copy number variation (CNV) detection [16]. The pathogenicity of all disease-attributable gene variants was reassessed based on the updated guideline of the American College of Medical Genetics and Genomics (ACMG) for molecular sequencing interpretation. Only patients with pathogenic or likely pathogenic mutations in the correct Mendelian inheritance pattern were recruited to the next step of the study, while cases with variants of unknown significance were excluded [17]. Patients with missing data or incomplete follow-up and irregular treatment were excluded from this study. Moreover, cases with secondary immunodeficiencies, more than one gene mutation, or without a definitive mutation were excluded.

### 2.4. Statistical Analysis

Qualitative variables were reported as absolute numbers and percentages. For quantitative data, median and interquartile ranges (IQR) were calculated. The patients were categorized and compared based on the phenotypical classifications provided via the 2021 update of the International Union of Immunological Societies (IUIS) [18]. The Mann–Whitney U, Wilcoxon, Chi-square, or Fisher exact test was utilized for the comparisons. All of the statistical analyses were performed using SPSS version 26.0 (IBM, Chicago, IL, USA). A *p*-value of less than 0.05 was considered statistically significant.

## 3. Results 

### 3.1. Population Characteristics

**Figure 1:**
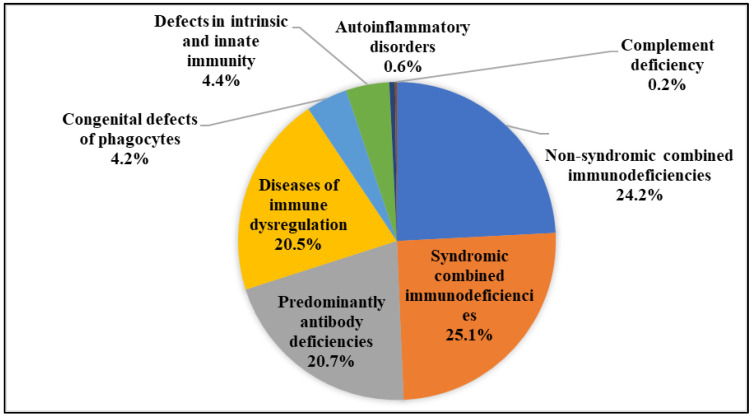
Classification of inborn errors of immunity in 521 Iranian patients with IEI.

A total of 521 monogenic IEI patients (37.6% female and 62.4% male) were assessed. About 72% (366 of 507) of patients were born to consanguineous parents. Most patients were categorized into syndromic combined immunodeficiency (syndromic-CIDs, 131 patients, 25.1%), non-syndromic CID (127 patients, 24.4%), predominantly antibody deficiency (PAD, 108 patients, 20.7%), and diseases of immune dysregulation (107 patients, 20.5%) (Figure 1). The commonly mutated genes were *ATM* (78 cases), *BTK* (70 cases), and *LRBA* (42 cases). Genetic defects in 54 patients were identified through targeted sequencing and 467 cases were solved using whole exome sequencing. Sanger sequencing was performed in all patients for confirmation of the mutations. Patients generally presented with infections (48.1%) at the median age of 1.0 (0.2–2.0) years. However, the diagnosis was mainly established 2.0 (0.2–4.9) years later at the median age of 4.0 (1.0–7.0) years. More than 74% of patients survived during the median of 8.7 (3.8–16.3) years of follow-up. The patients developed skin manifestations at a median (IQR) age of 2.0 (0.5–5.2) years. 

### 3.2. Cutaneous Involvements in the IEI Cohort

Skin disorders were among the clinical presentations of 43.5% (227 of 521) of all IEI patients. Additionally, 66 IEI patients (40.7% of 162 patients with available information) primarily manifested with cutaneous involvements. Of those patients, defects in intrinsic or innate immunity (60%) and non-syndromic CID (52.7%) mainly had early skin involvement (Table 1). On the other hand, skin involvements were a secondary feature in the majority of diseases of immune dysregulation (84.4%). Patients with cutaneous involvements were generally older at the time of IEI onset [1.0 (0.3–2.0) vs. 0.6 (0.2–1.9) years; *p* = 0.032] and diagnosis [5.0 (2.0–8.0) vs. 2.0 (1.0–6.0) years; *p* < 0.001] with a longer diagnostic delay [2.5 (0.2–5.0) vs. 1.1 (0.1–4.0) years; *p* = 0.005]. Consanguinity was more common among the patients who developed skin disorders (81.4% vs. 65.2%, *p* < 0.001)). We also compared the age of onset, diagnosis, and diagnostic delay in different phenotypical classifications in the presence and absence of cutaneous involvements. Similar to the overall data, patients with syndromic CID and cutaneous manifestations had a later age of onset [1.0 (0.5–2.0) vs. 0.2 (0–0.9) years; *p* = 0.001], age of diagnosis [5.0 (2.7–8.0) vs. 2.0 (0–4.0) years; *p* < 0.001], and diagnostic delay [3.0 (1.0–5.0) vs. 0.9 (0–3.8); *p* = 0.009] compared to the patients in the same category but without cutaneous features. Except for a longer diagnostic delay in patients with immune dysregulation and skin disorder than those without a skin disorder [5.0 (2.0–8.0) vs. 1.9 (0.4–5.2); *p* = 0.023], most parameters were not remarkably different.

**Figure 2:**
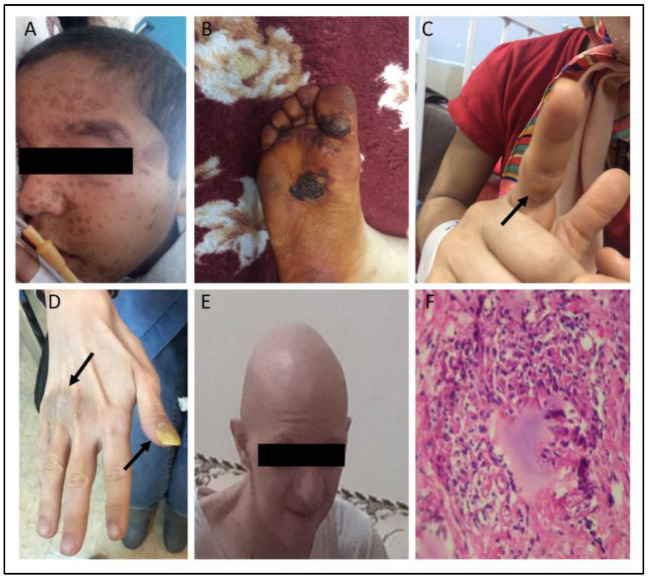
Skin manifestations of monogenic IEI patients. (**A**) severe VZV infections in DOCK8 deficiency. (**B**) Skin necrosis in MALT1 deficiency. (**C**) Skin wart refractory (indicated by the arrow) to treatment in CARMIL2 deficiency. (**D**) Onychomycosis (indicated by right arrow) and skin hyperpigmentation (indicated by left arrow) in STAT1 deficiency. (**E**) Alopecia universalis in LRBA deficiency. (**F**) Skin granulomatous inflammation lesion in NFKB1 deficiency.

These skin disorders were further categorized into infectious (129 of 227 cases; 56.8%) and non-infectious (188 of 227; 82.8%) manifestations. The dominant infective pathogens were detected in 78 patients, including fungi (37 out of 78; 44.9%), bacteria (28 out of 78; 35.9%), and viruses (15 out of 78; 19.2%). Non-infectious cutaneous presentations were also reported in these sub-groups of IEIs (Table 2). Skin malignancy was not detected in any IEI entities in our young cohort. Figure 2 represents some of the autoimmune and non-autoimmune cutaneous involvements in our patients. Of the immunologic parameters, IEI patients with skin disorders had significantly higher CD19+ cell percentages [12.0 (3.9–22.0)% vs. 6.9 (0.2–20.0)%; *p* = 0.002], IgA [19.5 (4.7–98.5) mg/dl vs. 10.0 (2.0–44.0) mg/dl; *p* = 0.004], IgG [605.5 (233.0–966.0) mg/dl vs. 240.0 (63.5–675.5) mg/dl; *p* < 0.001] and IgM levels [86.5 (35.0–186.5) mg/dl vs. 44.0 (14.0–144.5) mg/dl; *p* < 0.001] than those without skin involvements.

### 3.3. Dermatologic Characteristics of Different IEI Entities

These cutaneous manifestations have different frequencies in phenotypical IUIS classifications. These manifestations are more common among patients with syndromic CID, non-syndromic CID, diseases of immune dysregulation, congenital defects of phagocytes, defects in intrinsic or innate immunity, and PAD, respectively. The most common genetic defects in these patients were *ATM*, followed by *DOCK8* and *STAT3-AD (LOF)* (Table 3). Patients with *ATM* variants mainly presented telangiectasis, which is a characteristic feature in these patients, and autoimmune skin disorders, including vitiligo, psoriasis, and systemic lupus erythematosus. On the other hand, the majority of patients with *DOCK8* and *STAT3-AD (LOF)* mutations developed eczema, dermatitis, and viral skin infections.

**Figure 3:**
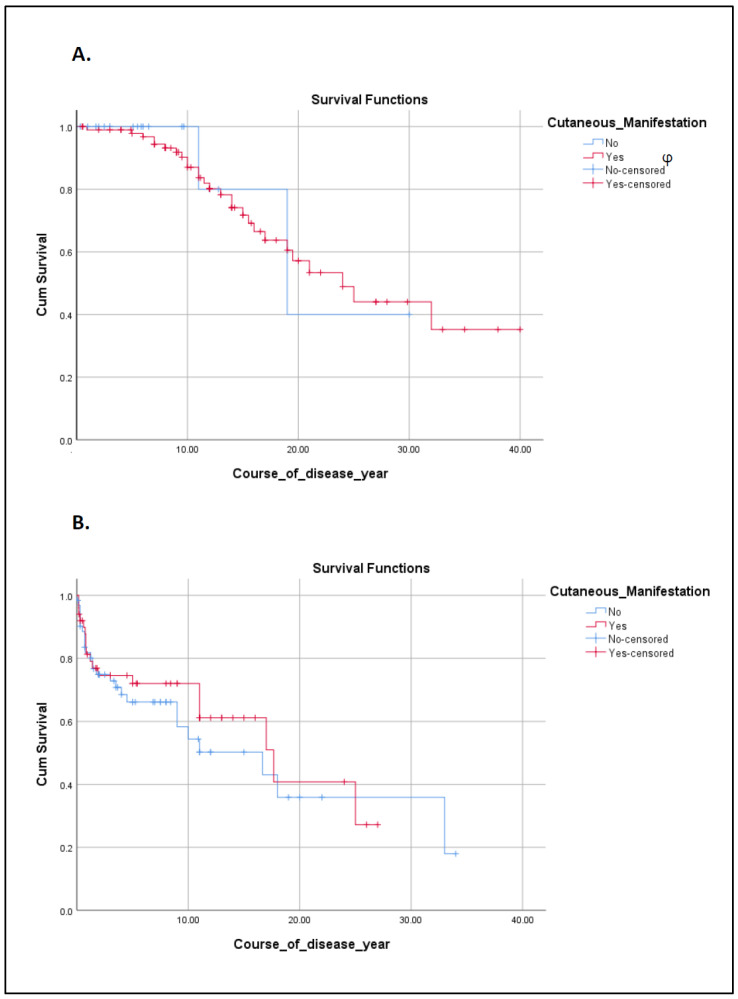
Kaplan–Meier survival curve of syndromic CID (**A**) and non-syndromic CID (**B**) patients with and without cutaneous involvements (φ censored refers to the subjects with shorter follow-up times compared to the cases with the highest survival rates and follow-up times).

The overall infection rate (the lowest rate in PAD patients) and the type of dominant pathogens (highest fungal infection in CID patients) were also significantly different among the IEI patients in different groups divided according to IUIS phenotypical classifications (*p* = 0.005 and *p* < 0.001, respectively, Table 2). Among non-infectious atopic cutaneous complications, urticaria was highly prevalent among patients with congenital defects of phagocytes (*p* = 0.021), with both cases bearing *RAC2* mutation. The frequency of eczema was also significantly different between the groups and patients; both syndromic and non-syndromic CID presented the highest susceptibility to this complication (*p* = 0.008). Vascular-related skin lesions (*p* < 0.001) and hypopigmentation (*p* = 0.003) were considerably more prevalent in syndromic CID and diseases of immune dysregulation, respectively. Table 3 provides detailed information on infectious and atopic manifestations in a patient carrying different gene mutations. Notably, the survival rate of IEI patients was affected by the presence of cutaneous involvements (*p* < 0.001). To generate a more accurate assessment, we compared survival in the two phenotypical groups with the highest number of patients. However, developing cutaneous disorders did not influence the survival rate of patients with neither syndromic nor non-syndromic CID (*p* = 0.846 and *p* = 0.539, respectively, as shown in Figure 3).

### 3.4. Autoimmune Cutaneous Manifestations in the IEI Cohort

Autoimmune cutaneous manifestations were documented in a small number of our patients (22; 9.6%) with cutaneous complications in types of alopecia (10 cases), vitiligo (7 cases), psoriasis (5 cases), and SLE (3 cases). Patients with autoimmune cutaneous disorders had a later disease onset [2.0 (0.3–7.7) vs. 1.0 (0.3–2.0) years, *p* = 0.038] and diagnosis [9.0 (5.0–15.0) vs. 5.0 (2.0–7.0), *p* = 0.001] compared to patients with other skin disorders. On the other hand, neither sex nor life status was different in patients with autoimmune cutaneous manifestations (*p* > 0.05). Alopecia and psoriasis were most common in patients with immune dysregulation (*p* = 0.001) and defects in intrinsic or innate immunity (*p* = 0.031), respectively. As shown in Table 3, the frequency of autoimmune cutaneous manifestations varied significantly among our patients, ranging from 2.9% in syndromic combined immunodeficient patients to 31.5% in patients with immune dysregulation (*p* < 0.001). Interestingly, the presence of autoimmune cutaneous complications significantly improved the survival rate among patients with cutaneous involvements (*p* = 0.19).

## 4. Discussion

Cutaneous involvements are an unappreciated aspect of clinical presentations in IEIs [19,20]. In the current report, we studied cutaneous changes in 521 monogenic patients with inborn errors of immunity from the Iranian registry. Most patients were categorized into syndromic CIDs followed by non-syndromic CIDs and PAD. Approximately 44% of our patients developed various skin disorders; 40.7% of those patients mainly presented features in form of skin infections. This prevalence of cutaneous involvement among IEI patients is lower than previous reports by Dhouib et al. and Al-Herz et al. [3,21] but similar to an earlier study from Iran [4]. Our IEI patients with skin manifestations were inclined to have a late disease onset and, particularly, disease diagnosis compared to patients without skin involvement. The median time for developing cutaneous manifestations was the approximate one-year interval from disease’s onset. Moreover, skin disorders altered the survival rate significantly, improving it by up to 20% in the first 10 years of follow-up. These patients also had a significantly higher consanguinity rate. Although the majority of patients with non-syndromic CID and defects in intrinsic or innate immunity primarily manifested with cutaneous involvements, these presentations were a secondary feature in most cases with immune dysregulation and PAD. Similar to Al-Herz et al. [21], the most common genetic defects in patients with skin diseases were *ATM*, *DOCK8*, and *STAT3-AD (LOF)*. Notably, the elevated level of CD19+ B cells and immunoglobulins among our patients with cutaneous involvements could result from the contribution of these immune mediators in developing eczema/dermatitis and autoimmune cutaneous manifestations in the patients [22,23]. Taken together, cutaneous disorders should be monitored in the course of IEI. Particularly, patients who carry *ATM*, *DOCK8*, and *STAT3-AD (LOF)* mutations are born to consanguineous parents and have elevated levels of CD19+ B cells and immunoglobulins.

We observed infectious cutaneous manifestations in 24.8% of all patients and 56.6% of patients with skin disorders, which is more common than observations recorded in previous publications from Colombia, Tunisia, Kuwait, and Mexico [2,3,21,24]. This result could arise from the higher number of patients in CID groups in our population and their potential susceptibility to infections. However, bacteria remained the main invading pathogen in our patients, followed by fungi and viruses, in accordance with other studies [21,24]. As anticipated [25], most fungal infections were noted in the group of cellular CIDs with abolished T-cell functions. However, contrary to former reports [3,21], bacterial infections were also predominantly detected in CID patients. Viral infections tended to be higher in patients with immune dysregulation, which could be explained as a result of the susceptibility to viruses in distinct gene mutations of this category [26].

Non-infectious cutaneous manifestations occurred in 82.8% of cases, especially in forms of vascular-related lesions and eczema. The high prevalence of vascular lesions in the overall cases, as well as the syndromic CIDs group, comes from the presence of telangiectasis as a rather characteristic feature in patients with *ATM* mutations [1]. Similar to infection, autoimmune cutaneous disorders were significantly different among the IUIS phenotypical groups. Interestingly, despite being a late-onset manifestation, the presence of autoimmune cutaneous complications raised the survival rate in our IEI patients. Therefore, it could be concluded that developing cutaneous autoimmunity in a patient might improve his/her prognosis. The prevalence of urticaria was remarkably higher in patients carrying the *RAC2* mutation, which is probably due to the defective mast cell functions in these patients [27]. Additionally, alopecia was common among patients with immune dysregulation, confirming a pre-disposition toward autoimmune diseases in this IEI category [28]. Furthermore, the higher prevalence of psoriasis in the intrinsic immunity defects (*IL12RB1* variant) group could be attributed to the role of IL-12 in psoriasis pathogenesis [29]. Comparable to almost all earlier observations [2,3,4,21,24], we recognized a greater frequency of eczema/atopic dermatitis in CID patients due to the consistent presence of eczema in hyper IgE syndrome and DOCK8 deficiency [18], that is, one of the main gene defects in our patients with cutaneous manifestations. Considering that hypopigmentation is a key feature in patients with Chediak Higashi, Griscelli type2, and Hermansky Pudlak type2 syndromes [1], it was highly observed in the patients with diseases of immune dysregulation.

Although managing the skin manifestations of IEI was not the focus of this study, it was observed that infections generally require both antimicrobials and antifungal agents, which are often prescribed prophylactically. Other non-infection skin disorders are mainly treated with topical and systemic immunosuppressive drugs [30,31,32]. This disease aspect needs to be mentioned in further investigations.

It should be noted that the retrospective nature of this study after IEI diagnosis could affect the results; thus, it is unknown whether more patients experienced cutaneous involvement as the first manifestation of their IEI. Moreover, some of our patients could represent cutaneous involvements later in their disease timeline, especially asymptomatic patients; this problem could also alter the results. Furthermore, most dermatological phenomena in IEI are not pathognomonic for a specific immunodeficiency, which is the reason why cutaneous involvements are mainly considered in the context of the natural course of their disease in cases with an established diagnosis of IEI. Thus, in addition to the disease diagnosis, the degree of involvement, response to treatment, course of the disease, and the prevalence of these skin manifestations should also be addressed in future studies.

## 5. Conclusions

In summary, cutaneous manifestations were detected in nearly 44% of Iranian patients with monogenic IEI. A considerable number of patients with cutaneous involvements developed these manifestations (predominantly skin infections) as the first manifestation of their IEI; this aspect was particularly noticed in patients with non-syndromic CIDs and phagocytic defects. The presence of skin disorders in IEI patients significantly delayed the diagnosis, which is generally established with a 3-year interval from the development of skin involvements. Cutaneous disorders, especially autoimmune cutaneous disorders, could improve the survival rate and prognosis of IEI patients. Bacterial infection was the main infectious cutaneous manifestation in our patients, followed by fungal and viral infections. Vascular lesions and eczema were the most prevalent non-infectious cutaneous presentation in our patients. Due to the unspecified skin manifestations in most IEI patients, more studies are required to attain a comprehensive clinical approach in this regard.

## Figures and Tables

**Table 1:** Distribution of skin disorders in 521 patients of different IEI groups.

Phenotypical Classifications	Presenting as a Primary Manifestation	Presenting as a Secondary Manifestation
Non-syndromic combined immunodeficiencies, *n* (%), (*n* = 55)	29 (52.7%)	26 (43.7%)
Syndromic combined immunodeficiencies, *n* (%), (*n* = 48)	21 (45.7%)	25 (54.3%)
Predominantly antibody deficiencies, *n* (%), (*n* = 16)	4 (25%)	12 (75%)
Diseases of immune dysregulation, *n* (%), (*n* = 33)	5 (15.6%)	27 (84.4%)
Congenital defects of phagocytes (number, function, or both), *n* (%), (*n* = 8)	4 (50%)	4 (50%)
Defects in intrinsic or innate immunity, *n* (%), (*n* = 5)	3 (60%)	2 (40%)

*n*; number.

**Table 2:** Associated skin disorders in different groups of IEIs.

Cutaneous Manifestations	Total (*n* = 227)	Non-Syndromic Combined Immunodeficiencies (*n* = 55)	Syndromic Combined Immunodeficiencies (*n* = 106)	Predominantly Antibody Deficiencies (*n* = 16)	Diseases of Immune Dysregulation (*n* = 37)	Congenital Defects of Phagocytes (Number, Function, or Both), (*n* = 8)	Defects in Intrinsic or Innate Immunity (*n* = 5)	*p*-Value
Infectious, *n* (%)	129 (56.8%)	45 (81.8%)	42 (39.6%)	7 (43.8%)	23 (62.2%)	8 (100%)	4 (80%)	<0.001
Fungal infection, *n*	35	19	4	1	8	1	2	<0.001
Viral infection, *n*	16	3	4	0	7	2	0	
Bacterial infection, *n*	44	13	15	4	6	4	2	
Non-infectious, *n* (%)	188 (82.8%)	41 (74.5%)	101 (95.3%)	11 (68.8%)	30 (81.1%)	3 (37.5%)	2 (40%)	<0.001
Eczema ^α^, *n* (%)	57 (33.5%)	23 (41.8%)	20 (40.8%)	0	12 (32.4%)	1 (12.5%)	1 (20%)	0.009
Vitiligo ^β^, *n* (%)	7 (4.1%)	2 (3.6%)	1 (2%)	1 (6.3%)	3 (8.1%)	0	0	0.686
Systemic lupus erythematous, *n* (%)	3 (1.8%)	1 (1.8%)	1 (2%)	1 (6.3%)	0	0	0	0.634
Alopecia, *n* (%)	10 (5.8%)	0	1 (2%)	1 (6.3%)	8 (21.6%)	0	0	0.001
Psoriasis, *n* (%)	5 (2.9%)	0	1 (2%)	2 (12.5%)	1 (2.7%)	0	1 (20%)	0.031
Ulcer, *n* (%)	4 (2.4%)	2 (3.6%)	2 (4.1%)	0	0	0	0	0.795
Hyperpigmentation, *n* (%)	7 (4.1%)	3 (5.5%)	4 (8.2%)	0	0	0	0	0.504
Hypopigmentation ^β^, *n* (%)	6 (3.5%)	0	0	0	6 (16.2%)	0	0	0.003
Vascular-related lesion, *n* (%)	77 (33.9%)	2 (3.6%)	74 (70.9%)	0	2 (5.4%)	0	0	<0.001
Atopic dermatitis ^α^, *n* (%)	13 (7.9%)	3 (5.5%)	5 (10.2%)	0	5 (13.5%)	0	0	0.570
Blister, *n* (%)	3 (1.8%)	1 (1.8%)	2 (4.1%)	0	0	0	0	0.786
Urticaria, *n* (%)	5 (2.9%)	0	1 (2%)	1 (6.3%)	1 (2.7%)	2 (25%)	0	0.020
Undefined skin disorders ^ɣ^	56 (32.9%)	14 (25.5%)	25 (51%)	5 (31.3%)	9 (24.3%)	2 (25%)	1 (20%)	0.068

*n*; number. ^α^ Atopic dermatitis is a type of eczema, whereas eczema refers to chronic general skin inflammation. ^β^ Hypopigmentation is a general term for skin discoloration; vitiligo is a skin disorder characterized with the presence of well-circumscribed, depigmented milky white macules devoid of identifiable melanocytes. ^ɣ^ Undefined skin disorders include “rash”, “skin lesion”, “xerosis”, and “erythroderma”.

**Table 3:** Distribution of different IEI and skin manifestations among 521 patients.

Phenotypical IEI Classifications	Total Number of Patients	Patients with Cutaneous Involvement	Skin Infections	Skin Atopic Manifestations	Skin Autoimmune Manifestations	Other Skin Manifestations
Non-syndromic combined immunodeficiencies	126	55 (44%)	45 (81.8%)	25 (45.5%)	2 (3.6%)	17 (30.9%)
T-B− SCID						
RAG1/RAG2 deficiency	21	9 (42.8%)	9	1	0	6
ADA deficiency	8	5 (62.5%)	5	1	0	2
Artemis deficiency	7	1 (14.2%)	1	0	0	1
Cernnunos/XLF deficiency	2	1 (50%)	1	0	0	0
T-B+ SCID						
No γδ T cells [CD3E(6), CD3D(1)]	7	3 (42.8%)(CD3E, CD3D)	3	1	0	0
JAK-3 deficiency	6	3 (50%)	3	3	0	0
gc deficiency	5	3 (60%)	2	0	0	1
Normal γδ T cells (PTPRC)	1	1 (100%)	1	0	0	-
Others						
CD40 ligand deficiency	26	3 (11.5%)	2	0	1	0
DOCK8 deficiency	25	21 (84%)	14	17	1	5
MHC class II deficiency [RFX5(2), RFXAP(1), RFXANK(4), CIITA(1)]	8	3 (37.5%)(RFXANK, RFXAP, RFX5)	3	1	0	1
IL-21R deficiency	2	0	-	-	-	-
IKBKB deficiency	2	1 (50%)	0	1	0	0
ZAP-70 deficiency	1	0	-	-	-	-
ICOS deficiency	1	0	-	-	-	-
CARD11 deficiency	1	0	-	-	-	-
DOCK2 deficiency	1	0	-	-	-	-
ITK deficiency	1	0	-	-	-	-
MALT1 deficiency	1	1 (100%)	1	0	0	1
Predominantly antibody deficiencies	108	16 (15%)	7 (43.8%)	1 (6.3%)	4 (25%)	5 (31.2%)
BTK deficiency (x-linked agammaglobulinemia)	70	7 (of 69 cases with data, 10.1%)	4	1	0	2
IGHM deficiency (Mu heavy chain deficiency)	13	3 (23.1%)	1	0	2	0
AID deficiency	9	0	-	-	-	-
PIK3R1 deficiency	5	1 (20%)	1	0	0	0
NFKB1 deficiency	3	1 (33.3%)	0	0	0	1
BAFF receptor deficiency	3	3 (100%)	1	0	1	2
BLNK deficiency	2	1 (50%)	0	0	1	0
Igα deficiency (CD79A)	2	0	-	-	-	-
TACI deficiency	1	0				
Syndromic combined immunodeficiencies	131	106 (80.9%)	42 (39.6%)	22 (20.7%)	3 (2.9%)	86 (83.5%)
ATM deficiency (ataxia telangiectasia)	78	74 (94.9%)	17	4	2	72
STAT3 deficiency (hyper IgE syndrome)	20	17 (85%)	14	12	1	7
DNMT3B	11	3 (27.2%)	2	0	0	1
ZBTBT24	6	3 (50%)	1	0	0	3
WAS deficiency (Wiskott-Aldrich syndrome)	11	7 (63.6%)	7	5	0	1
ARPC1B deficiency	2	1 (50%)	1	1	0	1
TTC7A deficiency (IEI with multiple intestinal atresias)	1	0	-	-	-	-
Purine nucleoside phosphorylase deficiency	1	0	-	-	-	-
IKBKG deficiency(NEMO deficiency)	1	1 (100%)	0	0	0	1
Diseases of immune dysregulation	107	38 (35.5%)	23 (60.5%)	14 (36.8%)	12 (31.5%)	13 (34.2%)
LRBA deficiency	42	12 (28.5%)	5	3	4	3
IL-10RB deficiency	13	3 (of 12 cases with data, 25%)	3	1	0	0
CD27 deficiency	9	2 (22.2%)	2	2	0	1
RAB27A deficiency (Griscelli Sd type 2)	6	3 (of 3 cases with data, 33.3%)	1	1	0	3
CD70 deficiency	5	2 (40%)	2	0	1	0
AIRE deficiency (APS-1)	4	4 (100%)	3	0	2	0
FOXP3 deficiency (immune dysregulation, polyendocrinopathy, enteropathy X-linked)	3	2 (of 2 cases with data, 100%)	2	2	1	1
CTLA4 deficiency	3	1 (33.3%)	1	0	1	0
XIAP deficiency (XLP2)	3	2 (66.6%)	1	1	2	1
AP3B1 deficiency (Hermansky Pudlak syndrome type 2)	2	2	0	0	0	2
STAT3 gain of function mutation	2	0	0	0	0	0
TNFRSF6 deficiency (ALPS syndrome)	2	1 (50%)	1	1	0	0
STXBP2 or Munc18-2 deficiency	1	0	-	-	-	-
SH2D1A deficiency (XLP1)	1	1 (100%)	0	1	1	0
UNC13D or Munc13-4 deficiency	1	0	-	-	-	-
ITCH deficiency	1	1 (100%)	1	1	0	1
PRF1 or perforin deficiency	1	0	-	-	-	-
PRKCD deficiency	1	0	-	-	-	-
RLTPR or CARMIL2 deficiency	1	1 (100%)	1	1	0	0
RASGRP1 deficiency	1	0	-	-	-	-
RIPK1 deficiency	1	-	-	-	-	-
TPP2 or tripeptidyl-peptidase II deficiency	2	0	-	-	-	-
LYST deficiency (Chediak-Higashi syndrome)	1	1	0	0	0	1
CD25 deficiency	1	0				
Congenital defects of phagocyte number, function or both	22	8 (38.1%)	8 (100%)	2 (25%)	0	2 (25%)
CGD [CYBA(1), NCF1(2), NCF2(1)]	4	1 (25%)(CYBA)	1	0	0	0
Elastase deficiency	4	1 (25%)	1	0	0	0
Kostmann disease	4	1 (25%)	1	0	0	1
Rac 2 deficiency	3	2 (66.6%)	2	2	0	1
Leukocyte adhesion deficiency I	2	1 (50%)	1	0	0	0
G-CSF receptor deficiency	1	0	-	-	-	-
VPS45 deficiency	1	1 (100%)	1	0	0	0
GFI 1 deficiency	1	0	-	-	-	-
G6PD deficiency	1	1 (100%)	1	0	0	0
Glycogen storage disease type 1b	1	0	-	-	-	-
Defects in intrinsic and innate immunity	23	5 (21.7%)	4 (80%)	1 (20%)	1 (20%)	1 (20%)
IL-12RB1 deficiency	19	3 (15.7%)	2	1	1	1
STAT1 deficiency	2	2 (100%)	2	0	0	0
TYK2 deficiency	1	0	-	-	-	-
IL-17RA deficiency	2	0	-	-	-	-
Autoinflammatory disorders	3	0	-	-	-	-
NLRP1 deficiency	1	0	-	-	-	-
PLCG2 deficiency (familial cold autoinflammatory syndrome)	1	0	-	-	-	-
MVK or mevalonate kinase deficiency	1	0	-	-	-	-
Complement deficiencies	1	0	-	-	-	-
FCN3 or ficolin 3 deficiency	1	0	-	-	-	-

## Data Availability

The data presented in this study are available on request from the corresponding author. The data are not publicly available due to ethical considerations.
